# Liposarcoma of the Arygepiglottic Fold: Teaching point: A mosaic tumor pattern mixing fatty and non-fatty enhancing components suggests liposarcoma, even in very rare locations such as neck spaces

**DOI:** 10.5334/jbsr.1868

**Published:** 2019-12-20

**Authors:** Sanaa Jamali, Pascal Van Eeckhout, Sandra Schmitz

**Affiliations:** 1 Cliniques Universitaires Saint Luc, BE

**Keywords:** Head and Neck cancer, Liposarcoma, Hypopharynx, CT scanner, Magnetic Resonance Imaging

## Abstract

Liposarcoma of the larynx and hypopharynx is very rare. Computed tomography (CT) and magnetic resonance imaging (MRI) examinations may synergistically disclose suggestive features of a fat-containing tumor with non-fatty enhancing sub-areas. Diagnosis relies on histological examination of a biopsic specimen. This rare pathology should ultimately be kept in mind when dealing with an laryngeal/hypopharyngeal mass.

## Introduction

Liposarcoma is the most common form of soft-tissue sarcoma, most frequently found in limbs and the retroperitoneum. Laryngeal and hypopharyngeal localizations are extremely rare; 40 cases have been reported in the literature up to now, three of which were of the dedifferentiated (DDL) histological subtype [2,3]. We report a case of DDL liposarcoma arising from the supraglottic side of the arygepiglottic fold.

## Case History

A healthy 67-year-old man presented with a four-month history of progressively worsening dysphonia and breathing discomfort. Neck palpation was unremarkable. Flexible laryngoscopic examination (not shown) revealed a polypoid and pedunculated mass of the right arytenoid and aryepiglottic fold.

Contrast-enhanced (CE) computed tomography (CT) demonstrated a fat-containing tumor arising from the right aryepiglottic fold. The lesion appeared well delineated with a mixed pattern. Magnetic resonance imaging (MRI) was performed after CT to better characterize the lesion and more precisely delineate tumor boundaries before surgical work-up.

**Figure 1:**
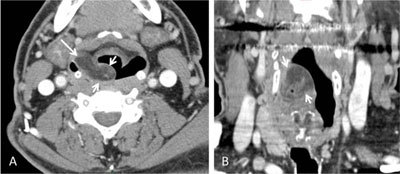
Contrast-enhanced CT work-up. Axial **(A)** and coronal **(B)** contrast-enhanced CT views show a well delineated fat-containing mass arising from the right ary-epiglottic fold (between arrows).

**Figure 2:**
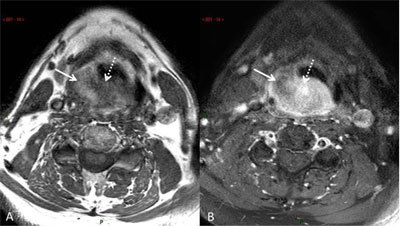
Contrast-enhanced MR work-up. Axial transverse T1-weighted magnetic resonance (MR) images in similar slice location. **(A)** Unenhanced section, showing coexistence of areas with fatty-like high signal intensity (arrow) and low signal intensity (dotted arrow). **(B)** Contrast-enhanced section with fat suppression showing decreased signal intensity (arrow) of the fatty areas, together with strong enhancement (dotted arrow) in areas disclosing low fat content on pre-contrast images (see 2A).

Fatty areas seen on CT (Figure 1A and 1B) expectedly disclosed high signal intensity on pre-contrast T1-weighted images (Figure 2A). Post-contrast T1-weighted images with fat suppression (FatSat) confirmed the mixed pattern of the tumor with darkened fatty areas strongly contrasting with enhancing areas without fatty content (Figure 2B).

**Figure 3:**
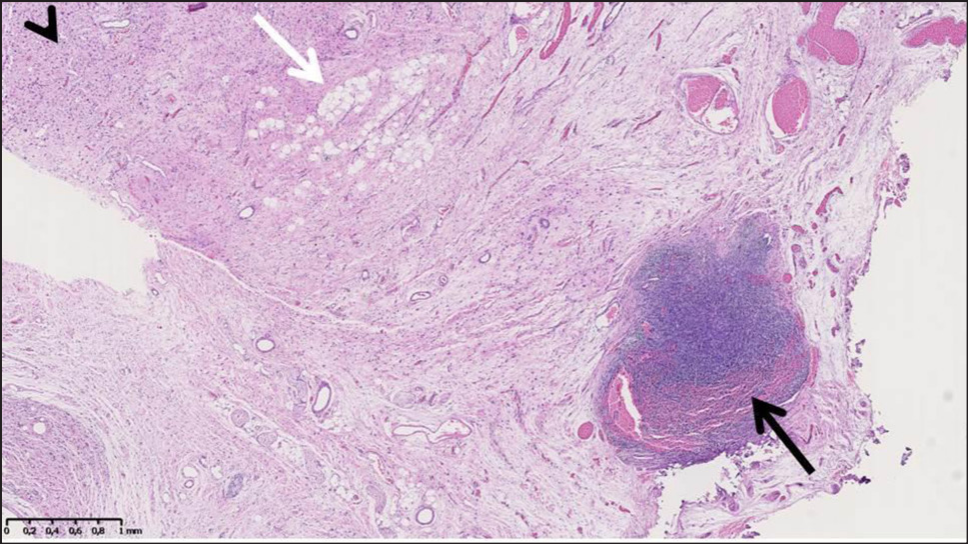
Histopathological examination of resected specimen. Lesion was a mosaic of areas of low- (arrowhead) and high-grade (black arrow) dedifferentiated liposarcoma (DDL) and well-differentiated liposarcoma (WDL) (white arrow). MDM2 gene amplification was assessed on both DDL and WDL areas. This focused view underestimated the true 90% proportion of WDL and DDL.

Supraglottic laryngectomy was carried out with clinically uneventful post-operative period, and completeness of the resection on MRI (not shown). Anatomopathological examination of the resection specimen demonstrated a large proportion of well-differentiated liposarcoma with a small areas of dedifferentiated liposarcoma, both with the MDM2 gene amplification (Figure 3).

## Discussion

Four histological subgroups of liposarcomas are described by the World Health Organization (WHO) classification: (i): differentiated (WDL); (ii): myxoid; (iii): pleomorphic; and (iiii): dedifferentiated (DDL). DDL is defined as non lipogenic sarcoma of variable (from low to high) histologic grades and may occur either as recurrence of WDL or arise *de novo*. As in our patient, WDL and DDL may share similar genetic signatures (e.g., a high level amplification of MDM2) [1]. Laryngeal and hypopharyngeal liposarcoma is an extremely rare condition with only three cases of the DDL histological subtype reported in the international literature [2,3,5].

Liposarcomas are well-delineated masses on CT and MRI. The presence of fat densities or signal intensities within the tumor is the main clue to the radiological diagnosis. Fatty content is a marker of WDL. ‘Mosaic’ appearance of the tumor may reflect the coexistence of lipogenic WDL and non lipogenic low- to high-grade DDL areas. In our patient a large proportion of WDL and small proportion of DDL was found, thereby explaining the prominence of fatty density/signal intensity on imaging. Mild enhancement is usually observed on post-contrast T1-weighted images, more conspicuously using fat saturation (Figure 2B).

Tumor extension is best assessed by CT or MRI, but the two modalities may act synergistically, with fat-suppressed post-contrast T1-weighted images better disclosing enhanced high-grade DDL areas embedded within fatty WDL that can be seen on CT and pre-contrast T1-weighted MRI. Mapping of such high-grade DDL areas is crucial both for biopsy targeting and overall treatment strategy. Our case illustrates a lipomatous tumor of overall pseudo-benign fatty appearance but containing small foci of high-grade tissue best depicted on post-contrast FatSat T1-weighted images.

Resection is the standard treatment option for HN liposarcomas through either external or endoscopic approach [4]. Conservative excision strategies are valuable treatment options even if recurrence rate is high in this condition. In recurring cases, total laryngectomy may be considered depending on disease extension and previous resection modalities. Systematic neck dissection is not indicated because the rate of nodal metastases is low [4]. Efficacy of postoperative radiation therapy has not been demonstrated so far.

## Conclusion

A mosaic tumor pattern mixing fatty and non-fatty enhancing components should trigger the diagnosis of liposarcoma even in very rare locations such as neck spaces.
